# Residual Properties in Damaged Laminated Composites through Nondestructive Testing: A Review

**DOI:** 10.3390/ma14164513

**Published:** 2021-08-11

**Authors:** Carlo Boursier Niutta, Andrea Tridello, Davide S. Paolino, Giovanni Belingardi

**Affiliations:** Department of Mechanical and Aerospace Engineering, Politecnico di Torino, 10129 Turin, Italy; Andrea.Tridello@polito.it (A.T.); Davide.Paolino@polito.it (D.S.P.); Giovanni.Belingardi@polito.it (G.B.)

**Keywords:** laminated composites, nondestructive techniques, damage severity assessment, residual properties, damage index

## Abstract

The development of damage tolerance strategies in the design of composite structures constitutes a major challenge for the widespread application of composite materials. Damage tolerance approaches require a proper combination of material behavior description and nondestructive techniques. In contrast to metals, strength degradation approaches, i.e., the residual strength in presence of cracks, are not straightforwardly enforceable in composites. The nonhomogeneous nature of such materials gives rise to several failure mechanisms and, therefore, the definition of an ultimate load carrying capacity is ambiguous. Nondestructive techniques are thus increasingly required, where the damage severity is quantified not only in terms of damage extension, but also in terms of material response of the damaged region. Based on different approaches, many nondestructive techniques have been proposed in the literature, which are able to provide a quantitative description of the material state. In the present paper, a review of such nondestructive techniques for laminated composites is presented. The main objective is to analyze the damage indexes related to each method and to point out their significance with respect to the residual mechanical performances, as a result of the working principle of each retained technique. A possible guide for future research on this subject is thus outlined.

## 1. Introduction

Nondestructive techniques (NDTs) aim at determining whether a structure will reliably perform in-service in the presence of flaws. In this regard, considerations on the flaw position, extension, or relative distance are usually required. More specifically, Sohn et al. [1] stated that the damaged state of a component can be described by answering the following questions:

1. Is there damage in the system (existence)?
2. Where is the damage in the system (location)?
3. What kind of damage is present (type)?
4. How severe is the damage (severity)?
5. How much useful life remains (prognosis)?

The damage state description is a five-step process where, following the order of the questions, the knowledge about the damage is increasingly acquired.

The concept of damage tolerance has also introduced new mechanical design strategies. In particular, fracture mechanics, which investigates the stress fields in the crack front and adopts energy-based criteria for the crack growth, has provided tools for addressing design problems in metallic structures. For metals, damage tolerance is established in terms of sufficient strength in the presence of cracks. The extension of the cracks defines the severity of the damage.

In the case of composite materials, the assessment of damage severity is not always unambiguous, and strategies for damage tolerant design still lack. Composites present several failure modes and flaw types which differently affect the mechanical performance. Anomalies are likely to arise during the manufacturing, such as matrix defects (i.e., voids, resin-rich zones, uncured matrix), fiber defects (i.e., fiber misalignment, unimpregnated or broken fibers), and interface defects (i.e., poor fiber/matrix or laminae bonding). During the service life, the composite component can experience overloads, impacts, and environmental conditions, which can degrade its mechanical performances. Such defects can act as crack initiators, causing premature failure, or can consistently affect the material stiffness, since irregularities in the material can drive the failure mechanism.

Without loss of generality, in the case of the unidirectional plies, when tensile loaded along the fiber direction, fibers firstly break singly or in clusters of two neighboring fibers, while in proximity of the final failure, clusters of four or more broken fibers can be observed [2]. The tensile strength is thus strongly influenced by the fiber integrity. Instead, misalignment of fibers consistently affects the failure stress in compression. Tensile loads in transverse direction, i.e., normal to the fiber direction, lead to fiber–matrix debonding, which is governed by a critical value of dilatational energy for the matrix [3]. The presence of defects (e.g., due to moisture) weakens the fiber–matrix interface, thus reducing the critical value of the dilatational energy. For a complete description of the failure mechanisms in unidirectional plies and laminated composites, the reader is referred to [4]. Analogous considerations on the influence of the microstructure on the mechanical response of the component can be argued for other composite typologies, e.g., woven based laminates. In addition, the combined effect of the different damage modes is still far from being established with robustness [4]. Analogously, the combination of loading conditions introduces further uncertainties, and the driving failure mechanism is case-dependent [5,6].

Previous observations highlight the importance of a proper assessment of the actual health state of a composite component. In particular, the multitude of damage mechanisms combined with the limited knowledge of their evolution and interaction suggests the need of assessing the damage severity on the basis of the material response in the presence of damage or flaws. The usual definition based on the extension of the cracked area is not sufficient for composites, and residual strength approaches typical of metals are not straightforwardly enforceable [7]. Indeed, the large variety of failure mechanisms differently affects the residual strengths of the material. For example in the case of impact damage, the presence of delamination cracks does not affect the tensile strength, as shown by Abrate [8]. Therefore, the assessment of a residual strength in composites does not represent a consistent metric for damage severity. It is also worth noticing that the assessment of the residual strengths of composites correlated to a specific damage state of the material still represents a major challenge for researchers [7].

In this paper, the NDTs able to provide a quantitative description of the material state are revised. As the paper is particularly focused on NDTs, structural health monitoring (SHM) methods, such as the acoustic emission, will not be discussed. Further, other NDTs unable to quantify the damage severity, such as the tomography, will not be addressed. The reader is referred to [9] for an exhaustive and recent review. Differently from the work [9] and other reviews on the same topic, e.g., [10,11], the main objective of the present paper is to analyze the damage indexes related to each method and to point out their significance with respect to the residual mechanical performances. Ideally, damage indexes in composites inspection should be able to characterize the local material condition, thus identifying the damage severity, while accounting for the directional dependency of composites. A proper NDT for composites should fully characterize the material response in presence of damage, i.e., the residual mechanical performance and its directional dependency should be determined. Under these considerations, a critical review of the current NDTs also outlines a possible guide for future research on this subject.

For the sake of clarity, in the following “Authors” with the capital letter will refer to the authors of the original paper, whereas “authors” will refer to the authors of the present paper.

The paper is organized as follows: the retained NDTs are described and discussed, one by one. In particular, for each considered technique, the working principle is briefly presented, in order to introduce the significance of each damage index. Thereafter, results reported in the literature are shown and commented on. Finally, the damage metric related to each technique is analyzed. Firstly, acoustic wave-based methods, i.e., ultrasonics and acousto-ultrasonics will be addressed. Then, global and local vibrational methods and a nondestructive impact-based technique, called Detecting Damage Index, will be addressed, followed by optical methods and infrared thermography. Finally, resistivity-based technique which is specific of conductive composites will be discussed.

A specific consideration is required for the terahertz technique, whose application on composite structures is rapidly increasing. Concerning the material response, the terahertz technique provides information on the optical properties of the material, in particular the refractive index and the absorption coefficient, calculated from the phase of the acquired signal in the terahertz test. The variation of these parameters is adopted to establish the presence of damage, its location and extension [12,13]. Theoretically, these parameters can be used to quantitatively assess the damage severity. In this regard, Stoik et al. [14] have shown a significant variation of the refractive index in the whole inspected frequency range in burned composite samples. More recently, Kim et al. [15] have shown a consistent reduction of the signal amplitude in correspondence of the damaged zone. It is, however, worth noticing that the use of optical parameters for damage severity characterization is not devoid of ambiguity. Similarly to the micro-computed tomography, damage mechanisms as the matrix plasticization cannot be detected through optical inspections. Further, to the best of the authors’ knowledge, damage indexes associated to the material response, i.e., refractive index and the absorption coefficient, have not been reported in the literature, as well as the significance of the variation of these parameters has not been discussed. For these reasons, terahertz will not be considered in the following.

## 2. Waves Methods

In this section, wave methods are analyzed. In particular, these methods are based on ultrasonic, acousto-ultrasonic, and nonlinear acoustic techniques. The nonlinear acoustic technique, however, is not able to provide a quantitative evaluation of the damage state [16]. Indeed, also the local defect resonance methodology [17,18], where the component is excited at a frequency such that the damaged region is forced in resonance and so the displacements are much higher than the surroundings, requires that the material properties and the location of the damage are known. As such, the nonlinear acoustic method will not be further discussed.

Both ultrasonic and acousto-ultrasonic methods adopt high-frequency waves to inspect the material. Waves are generally induced by means of piezoelectric transducers, which convert the input signal from electrical to mechanical and conversely for the output signal. The output signal can refer to the transmitted waves when two transducers are adopted, or, in alternative, to the waves reflected by the back surface. In the latter case only one transducer is employed. In order to obtain reliable results, the coupling between the inspected material and the transducers has to be carefully realized. Ideally, the coupling medium is isotropic and indefinitely deformable [9]. Fluids, such as water, oil or air are therefore suitable. Water is among the most used coupling media, where the component and the transducers are immersed in a tank of water for the nondestructive inspection. The consistent coupling guaranteed by the water permits a complete scan of the component, by moving the transducers with respect to the investigated material. Air can also be employed, especially in those applications where the component cannot be immersed in a tank of water due to dimensional or contamination constraints [19]. The main drawback of the air coupling is the low frequency range that can be adopted for the investigations (from 50 to 500 kHz), as a result of the very different propagation velocities of waves in air and in the solid media. As a consequence, the depth of investigation of the material is limited in air-coupled ultrasonic technique.

### 2.1. Ultrasonics

Ultrasonic technique exploits volume waves to investigate the material quality. In particular, the reflection, the transmission or the backscattering of longitudinal and shear waves is detected. Pulsed waves cover the range from 20 kHz to 1 GHz, even though in practical applications the most adopted frequencies are within the range from 0.5 MHz to 10 MHz. Ultrasonics inspections are characterized by three investigating modes: A-scan, where punctual measurements are done to assess damage depth, B-scan, where several A-scan measures allow to obtain information as a function of the position and C-scan, which is the most adopted scheme. C-scan consists of several B-scan style paths, which allow to map the component. Other operative modes, such as D-scan, G-scan, and X-scan, have been proposed in the literature, even though less popular [9]. According to [20], ultrasonic testing is currently the only nondestructive technique leading to certification. Indeed, by combining normal and oblique pulse echoes, a highly detailed volumetric image of complex damage states can be produced, as shown by Aymerich et al. [21] for impacted composite laminates.

Ultrasonics have been also adopted to characterize the residual elastic properties in damaged composites [22,23]. Indeed, the propagation velocity, also called phase velocity, of the longitudinal and shear waves in the material depends on its elastic constants. An eigenvalue problem can be thus formulated as follows [24]:

(1) $$det\left(C_{ijkl}n_{j}n_{l}-\rho V^{2}\delta_{ik}\right)=0$$

where $C_{ijkl}$ are the stiffness tensor coefficients, $n_{j}$ and $n_{l}$ are the components of the unit vector n describing the propagation directions of the wave, ρ the material density, V the phase velocity and $\delta_{ik}$ the Kronecker operator. From the measurement of the phase velocities in each direction, the corresponding coefficients of the stiffness matrix can be determined through an optimization scheme based on the Levenberg–Marquard algorithm [24]. A damage index can be thus defined and calculated for each coefficient as:

(2) $$D_{ij}=1-\frac{C_{ijkl}}{C_{ijkl,0}}$$

where $C_{ijkl,0}$ and $C_{ijkl}$ are the initial and current stiffness coefficients. The methodology adopts a specifically designed device able to rotate with respect to the investigated specimen, in order to measure the phase velocities for different orientations [25]. In particular, the phase velocities are determined from the time-of-flight recorded by the transducers. The device, the composite specimen and the transducers are immersed in a tank of water.

**Figure 1:**
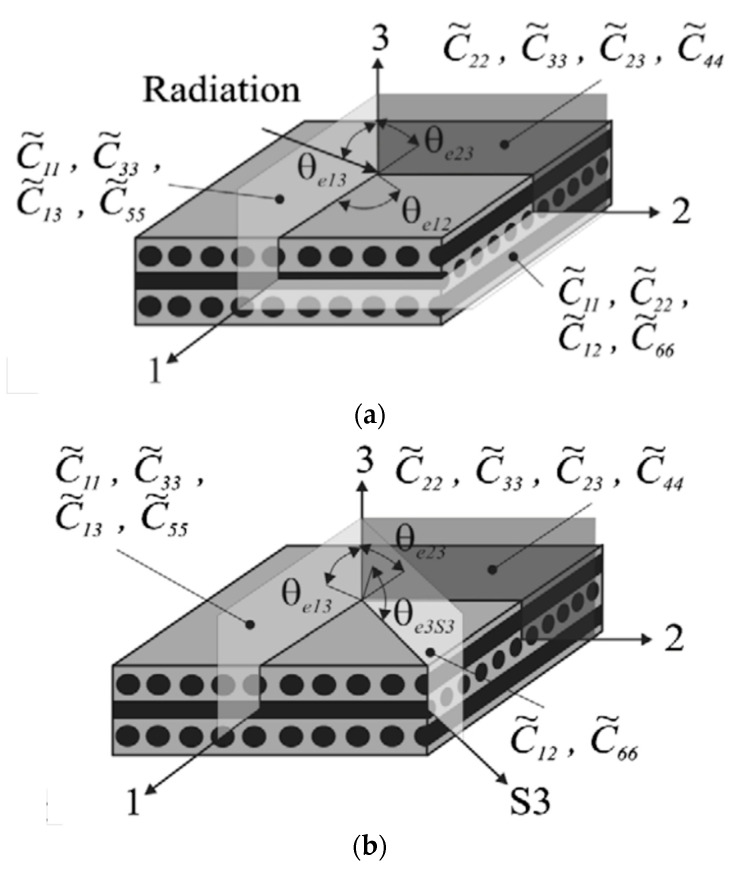
Determination of stiffness matrix coefficients by measurement in (**a**) symmetry planes and (**b**) non-symmetry planes [26].

Regarding orthotropic materials, nine measurements of the wave velocity are required to get a complete characterization. These must be determined along the axes of orthotropy of the material, as shown in Figure 1 taken from [26].

However, as the thickness is usually rather small in composite components, velocities in the plane 1–2 of Figure 1a cannot be measured, according to [24]. In particular, transverse waves are generated only for incident angles of the transmitted wave larger than 5°, according to [22]. The requirement of an incident angle combined with the typical small thickness of the composite components does not allow the propagation of the transverse wave in the plane 1–2. The Authors thus proposed to perform measurements in a non-symmetry plane, as shown in Figure 1b.

Measurements in non-symmetry planes are, however, controversial due to practical problems [26]. Indeed, the determination of the time-of-flight data is complicated by the severe distinction between the phase velocity and the group velocity, i.e., the velocity of propagation of the wave packet, in non-principal planes [24]. Further, it is worth noticing that the methodology assumes an orthorhombic symmetry in the material [25]. Therefore, apart from the special case of cross-ply symmetric laminates, material constants of angle-ply laminates cannot be determined.

### 2.2. Acousto-Ultrasonics

Acousto-ultrasonics is recognized as one of the most promising techniques for the quantitative assessment of damage in composites. The name “Acousto-ultrasonics” suggests the combination of acoustic emission and ultrasonic methods. As stated by Vary [27], “Acousto-ultrasonics” can be seen as the contraction of “Acoustic emission simulation with ultrasonic sources”. Indeed, the technique intends to inspect the structure by simulating the acoustic emission of waves, which typically accompanies plastic deformation and crack growth, through nondestructive ultrasonic waves.

More specifically, acousto-ultrasonic technique involves Lamb waves, also called guided waves. Mathematically, guided waves are the result of the partial differential equations governing the propagation of a wave within the material, where boundary conditions, i.e., the physical limits of the component, are retained [28]. Physically, Lamb waves can be seen as the interference of longitudinal and shear waves which satisfies the boundary conditions and occur in free plates with traction-free upper and lower boundaries.

**Figure 2:**
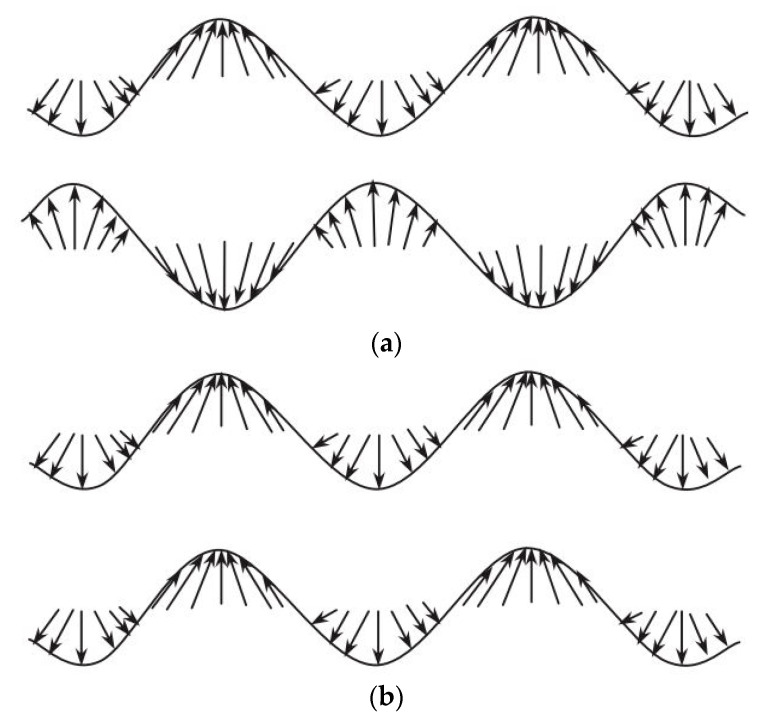
Symmetric (**a**) and anti-symmetric (**b**) Lamb waves [29].

Multiple propagating modes can be excited for the nondestructive inspection. Usually, the investigating frequency range is limited and only the first two fundamental modes are observed: the anti-symmetric mode, called $a_{0}$ and the symmetric mode, called $s_{0}$, which are shown in Figure 2, taken from [29].

The suitable frequency range can be determined through the so-called dispersion curves, as in the program developed by Pavlakovic et al. [30]. Both anti-symmetric and symmetric modes can be adopted for damage detection. Lammering et al. [31] suggest that, in antisymmetric mode, surface regions transmit most of the energy and thus are mainly suitable for surface damage. However, it is worth noticing that acousto-ultrasonics can inspect the entire cross-sectional area [29].

Thanks to the limited natural attenuation, Lamb waves can easily propagate in the component, allowing for the inspection of large areas. Differently from the ultrasonic technique, a line rather than a point is interrogated, through two transducers: the sending and the receiving ones. The receiving transducer monitors both transmitted waves and waves reflected to the sending transducer. Reflections occur in correspondence of the boundaries, i.e., component borders and flaws. The analysis of the reflected waves, as well as of refraction and mode conversion phenomena, provides information on the damage position and size.

In laminated composites, in addition to the Lamb waves, i.e., longitudinal and vertical shear modes, a transverse in-plane shear motion was firstly observed by Love in 1911 and then confirmed in other experimental and numerical studies [29]. This mode, also called horizontal shear mode, particularly complicates the analysis of results. Indeed, for waves propagating in the fiber direction and in the two directions perpendicular to it, the longitudinal and shear waves are uncoupled. In all the other directions, longitudinal and shear waves are coupled, as a consequence of the directional dependency of the waves propagation. As such, the analysis of laminated composites through acousto-ultrasonic method is particularly complicated, apart from the special case of cross-ply symmetric laminates.

Several damage indexes have been proposed in the literature, where analysis of results is performed both in time and frequency domains. They can be classified in two categories. In the first, velocity measurements are retained for the assessment of residual elastic properties. Similarly to the ultrasonic technique (Cf. Section 2.1), the time of flight is measured and compared to the reference (undamaged) state. The whole received signal can be also used to define a damage index. These indexes are based on the time domain description of the signal and retain the peak-to-peak amplitude, the root mean square or the variance of the baseline and actual signals [32].

In the specific case of symmetric cross-ply laminates, the propagation velocity of the $s_{0}$ mode waves can be expressed as [33]:

(3) $$V=\sqrt{\frac{E_{11}}{\rho \left(1-\nu_{12}\nu_{21}\right)}}$$

where V is propagation velocity, $E_{11}$ the longitudinal Young’s modulus, ρ the mass density and $\nu_{12}$ and $\nu_{21}$ the in-plane Poisson’s coefficients. The propagation velocity is approximated as $\sqrt{\frac{E_{11}}{\rho }}$, which allows to define a damage index by comparison with the reference undamaged state as:

(4) $$D=1-\frac{E_{11}}{E_{11,0}}$$

where $E_{11}$ and $E_{11,0}$ are the current and reference longitudinal Young’s modulus. In combination with a shear-lag analysis, the Authors were able to predict the crack density evolution in the off-axis plies [34]. They also correctly captured the stiffening and softening effects in the 0° and 90° plies, respectively [35,36]. The technique has been also adopted for damage assessment in the case of delamination induced by impact [37]. A mapping procedure that consists of systematic transducer movements on the composite plate allowed to locate and estimate the extension of a delaminated area. It is worth noticing that the assessment is related to the material comprised between the transducers [38,39], which makes the technique particularly suitable for extended damage, such as the subcritical matrix cracking typical of off-axis plies in laminates.

The second category of damage indexes adopts acoustic emission methodologies for the analysis of the results. In particular, measurements of the signal are expressed through the so-called Stress Wave Factor (SWF), which usually refers to the $s_{0}$ Lamb waves. The description of the SWF can be performed both in time and frequency domains. In its earliest definition, the SWF essentially consists in counting the peaks in the received signal which cross an assumed threshold voltage [40]. The threshold voltage is usually set at just above the noise level [27]. The pulses are generated at a chosen repetition rate “R” and the number of threshold crossings “C” is counted in the time interval “T”. The SWF is thus defined as the product RCT. This approach was indeed found to be related to the material strength [41].

As an alternative, Talreja proposed the definition of five SWFs in the frequency domain of the received signal [42]. The Author showed that the proposed SWFs were particularly sensitive to the relative orientation of the transducers with respect to the material, as a result of the material anisotropy. Therefore, a correlation between the SWFs and the local elastic properties was suggested, even though not explicitly articulated. Recently, Pillarisetti et al. [43] have shown that the same SWFs definition well correlates with the crack density in damaged composites. However, as stated by the authors, a correlation between local elastic properties and measured SWFs still needs to be established. In this regard, the nonphysical meaning of the SWFs does not help. In addition, it is worth noticing that the scatter and low replicability of signals demand for statistical analysis. As shown by Russel-Floyd et al. [44], when statistical methods are applied to account for the scatter of SWF readings, even impact damages of 15 mm diameter can be undetectable.

## 3. Vibrational Methods

Low-frequency vibration methods retain the modal characteristics of the structure to inspect its health state. In particular, the structural health monitoring arises from the observation that changes in the structural properties have consequences on the natural frequencies [45]. Several procedures have been proposed in the literature. These can be discerned in global or local, whether the investigation concerns the whole component or a circumscribed region.

### 3.1. Global Vibrational Analysis

In global methods, the vibrational response of the whole structure, i.e., displacement, velocity or acceleration, is measured through proper recording equipment. The signal is then processed with fast Fourier transform, in order to compute the resonant frequencies, the modal shapes and the damping parameters. Variations in the modal response of the system are then exploited to quantify the material degradation. Through comparison with the undamaged condition, damage severity is assessed in terms of residual properties or residual stiffness. Lifshitz and Rotem [46] firstly proposed the use of vibration measurements for damage detection. They particularly searched for changes in the dynamic moduli, which are related to shifts in the natural frequencies, to detect damage in elastomer specimens. These methods are also very attractive for interrogating the whole component rather quickly, eventually through a single measure. Low frequency vibration methods can be successfully applied for quality control [47,48,49].

However, as the global behavior is retained, only high-level damage or large defects, such as errors in the stacking sequence or incorrect fiber volume fraction can be detected. The capacity to locate local damages is significantly limited [38]. Zak et al. [50] performed experimental and numerical analyses of composite beams and plates. These structures were clamped at one side and delaminated at the other. Different ratios of delamination length with respect to the total structure length were considered. Results showed that for delaminations of length up to 30% of the total structure length, the reduction of the first resonant frequency was smaller than 5%. Similar values were obtained for higher modes. Further, they also showed that when the delamination moves away from the neutral plane, the drop of the natural frequencies is even smaller.

Further, vibration methods are inherently unable to localize the damage. Cawley and Adams [51] proposed a frequency based approach for damage localization. They showed that the ratio between the frequency shifts of modes i and j, $\frac{\Delta \omega_{i}}{\Delta \omega_{j}}$, is only function of the damage position. Even though attractive, the method lacks robustness, as the small reductions of natural frequencies consistently affect the detection. Analogous considerations can be done for the approach proposed by Hu and Wang [52]. The Authors retained the modal deformation energy, which depends on the modal shape, to localize the damage on an impacted plate. In particular, the plate is divided into subregions and, according to the modal shape, the modal deformation energy is calculated for each subregion. For a given modal shape, a damage index can be defined in each subregion as the ratio between the modal deformation energy before and after the impact. High values of the damage index for a given subregion indicate the presence of the damage and thus locates it. Apart from the tedious repetitions of the frequency measurement necessary to assess the modal shape, the small variations of the natural frequencies compromise the robustness of the methodology. When global vibration behavior is analyzed, the in situ anomaly is concealed by the surrounding intact material [53,54].

### 3.2. Local Vibrational Analysis

In contrast to global methods, local vibrational analysis concerns a circumscribed region of the component. As such, the presence of localized damage, such as in impact events, can be more easily highlighted. Apart from the so-called “coin-tap” test where an operator locally impacts the component through a coin and listens to the resulting sound and whose pure subjectivity does not allow extensive use, an innovative methodology based on the Impulse Excitation Technique (IET) has been recently proposed [55,56].

**Figure 3:**
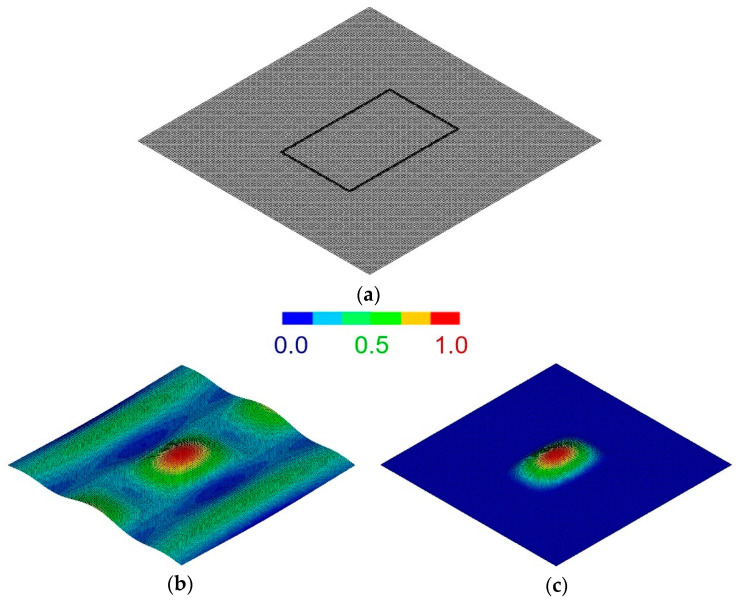
Comparison of local simply supported and clamped boundaries: (**a**) finite element model with highlighted path of constrained nodes; (**b**) modal analysis results with simply supported boundaries; (**c**) modal analysis results with clamped boundaries [55,56].

The technique is able to localize the vibrational response of the system by adopting the proper equipment. In particular, the boundaries of the region under investigation are nondestructively clamped. By clamping the boundaries of the region, the Authors showed that a vibrational mode which is specific of the retained region can be excited. The resultant first resonant frequency is thus only a function of the elastic characteristics of the inspected region. Figure 3, taken from [56], shows the results of a finite element modal analysis where simply supported (Figure 3b) and clamped (Figure 3c) boundaries are alternatively retained for the path of nodes highlighted in Figure 3a. When fully clamped boundaries are adopted, a specific mode which involves only the inspected region can be excited.

The methodology is based on the IET. An impulser is used to excite the plate and the resulting mechanical vibrations are measured through a microphone. The signal is then processed to provide the first fundamental frequency of the retained region. The technique is also shown sensitive to the material anisotropy, as different values of the resonant frequency are measured after rotating the clamping system with respect to the composite plate. The material constants are finally obtained from the measured frequencies through an inverse optimization algorithm based on Finite Element analysis.

**Figure 4:**
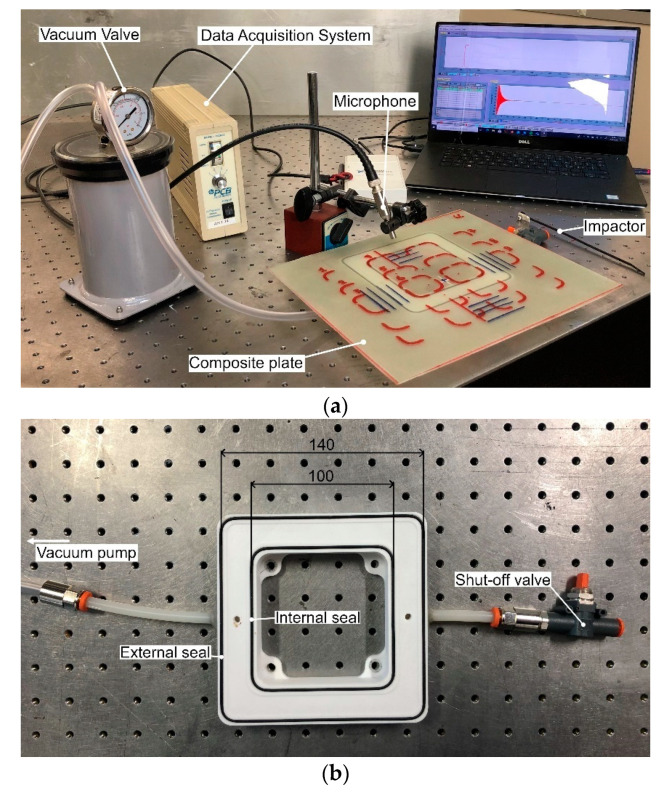
Experimental setup (**a**) and clamping device (**b**) of the local vibrational analysis technique.

The technique has been recently extended to the analysis of damaged laminates. In particular, as the observations performed within the nondestructive testing are usually limited to only the exposed surface of the component, an innovative clamping system has been proposed, which adopted a vacuum to obtain the clamping effect. As shown in Figure 4, the inspected region is framed by a vacuum chamber whose air is aspired through a vacuum pump. The frame chamber is delimited by internal and external rubber seals, which prevent any air escape.

Complementarily, a new analytical approach is derived in order to specifically determine the residual elastic properties of the damaged region. Thanks to the sensitivity to the material direction, the damage index can be defined in accordance with the investigated direction as:

(5) $$D_{ij}=1-\frac{E_{ij}}{E_{ij,0}}$$

where $E_{ij,0}$ and $E_{ij}$ are the initial and current material properties, respectively. The technique is validated on glass-fiber woven fabric laminates of six and eight layers damaged through impact.

## 4. Detecting Damage Index—Nondestructive Impacts

The energy absorption aptitude of composites in case of impact damage has been widely investigated in the literature [57]. At increasing impact energy, delamination, matrix cracking and fiber fracturing subsequently occur in the laminate. Energy balance approaches have also highlighted the damage accumulation mechanisms typical of impacted composites [58,59]. As plastic deformation of composites is very limited if not null, the impacting energy $E_{i}$ is dissipated both through elastic deformation and by fracturing. Net of the elastic energy $E_{el}$, whereby the impactor rebounds, the residual part of the incident energy is absorbed in creating large areas of fracture. Impact damages are thus strictly correlated to the absorbed energy $E_{abs}$, which can be in turn interpreted as a measure of the material damage [60,61].

**Figure 5:**
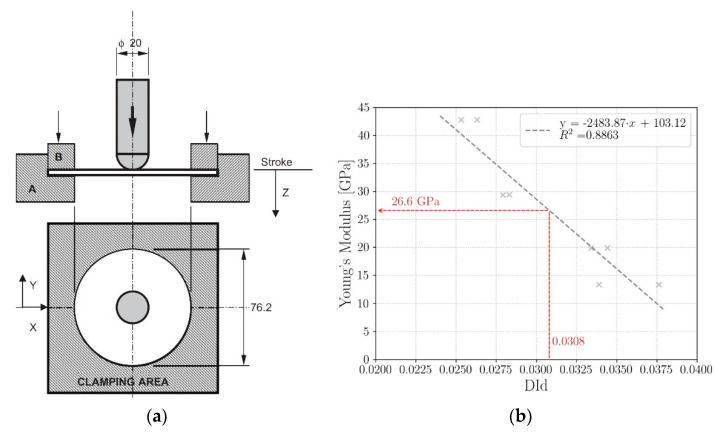
(**a**) Scheme of the testing fixture of the $DI_{d}$ technique [61]; (**b**) relationship between the residual elastic properties and the $DI_{d}$ parameter [64].

Based on these considerations, a nondestructive technique has been recently developed [62,63]. The methodology is called Detecting Damage Index ($DI_{d}$) and exploits nondestructive impacts to quantify the residual elastic properties in damaged composites. As the residual energy absorption aptitude is evaluated for a circumscribed region of the component, local elastic constants are assessed. Figure 5a shows the testing fixture. By clamping the boundaries through a properly provided uniform pressure, the impact response concerns only the circumferential region whose diameter is 76.2 mm.

The $DI_{d}$ parameter is defined as:

(6) $$DI_{d}=\frac{E_{abs}}{E_{th}}\cdot \frac{s_{MAX}}{s_{QS}}$$

In Equation (6), $E_{th}$ is the so-called threshold energy, which is defined as the impact energy at which the reduction of local elastic properties is less than 5%. An impact at the threshold energy can be considered as nondestructive for the material. This value is a material property which has to be identified with a preliminary characterization. The absorbed energy $E_{abs}$ is calculated as the area included within the load–displacement curve. Finally, $s_{MAX}$ and $s_{QS}$ represent the maximum displacement recorded during the test and the displacement value recorded in quasi-static perforation test at the instant when the perforation takes place and the resisting force becomes constant, respectively. The ratio between the maximum displacement and the value recorded in quasi-static perforation test allows to discern between the penetration and the perforation [61].

The $DI_{d}$ technique thus requires a rather extended preliminary characterization activity which permits to identify, firstly, the threshold energy $E_{th}$ and, secondly, the correlation between residual elastic properties of the material and the $DI_{d}$ parameter. In addition, the preliminary campaign is preceded by a quasi-static perforation test to compute the displacement value $s_{QS}$ at the instant when the force becomes constant.

The preliminary experimental campaign consists of two sets of impact tests: the so-called “single impact set” and “double impact set”. In the single impact set, material plates are impacted at increasing impact energy levels in order to increasingly damage the composite. Tensile tests are then performed on specimens cut from the impacted plates around the damaged area. In order to determine the residual elastic properties, longitudinal strains are measured through an extensometer mounted near the damaged zone. The tensile tests allow to correlate the residual elastic properties to the impact energy. From this correlation, the so-called threshold energy $E_{th}$ is evaluated.

The double impact set involves two impact tests on each plate. A new set of composite plates is initially impacted at increasing energy levels and then impacted again on the damaged area at the threshold impact energy $E_{th}$. The damage index parameter $DI_{d}$ is thus calculated according to Equation (6). The double impact set allows to correlate the $DI_{d}$ parameter to the impact energy. Combining this information with the results of the single impact set, where the residual elastic properties are correlated to the incident energy, the residual elastic properties can be expressed as a function of the $DI_{d}$ parameter, as shown in Figure 5b.

The dependency of the material properties on the direction can be accounted by repeating the tensile tests after having cut specimens in the desired directions from the single impact set. The damage index can be therefore defined as:

(7) $$D_{ij}=1-\frac{E_{ij}}{E_{ij,0}}$$

where $E_{ij,0}$ and $E_{ij}$ are the initial and current material properties in the retained direction.

The effectiveness of the $DI_{d}$ technique has been shown on a carbon/epoxy laminate in combination with the finite element method [64]. The laminate plate has been firstly damaged through repeated four-point bending tests. Then, the $DI_{d}$ technique has been adopted to map the residual elastic properties on the damaged plate. The variation of the Young’s modulus is accounted in the finite element model of the laminate subjected to bending test through a continuous polynomial curve. The resulting experimental and numerical force–displacement curves are finally compared, proving the accuracy of the technique. The ability to locally assess the residual elastic properties is particularly convenient when the damage is localized within the component, such as in the case of impact events. However, even though the $DI_{d}$ methodology does not involve expensive devices, an extensive preliminary experimental campaign is required.

## 5. Optical Methods

Optical methods are non-contact techniques aiming at measuring the displacements of the structure surface under the application of external loads. Generally, in correspondence of defects, displacement gradients are more rapid, thus allowing the damage localization. Further, the quantitative assessment of the full-field strains allows to compute scalar damage variables. In order to determine the surface displacement, speckle patterns are generated through either laser light or manual spraying, depending on the different measurement technique. Both static and dynamic loads can be applied: the structure is usually loaded through vacuum systems, even though thermal loads or vibrations can be also adopted [65]. Example of the optical methods are the shearography, the speckle interferometry and the digital image correlation (DIC).

In shearography, the speckle pattern is obtained by illuminating the tested object with a laser source. A shearing device and a polarizer are used to align the non-parallel light beams reflected by the specimen and to enable their interference, respectively. As the object surface is optically rough, the interference of two shared beams will result in a speckle pattern embedded in the shearographic image [66]. When the component is deformed, the speckle pattern is modified, and the strain field is evidenced. Garnier et al. [20] have successfully applied this technique to specimens with variable geometry. Speckle interferometry adopts a similar process for the generation of the speckle pattern. The object is illuminated with a laser beam and a speckle pattern is firstly assumed in the unstressed state. Then, the speckled image is made to interfere with the reference state in order to establish displacement gradients. In particular, phase changes between the reference and the current intensity distribution of the speckle pattern are used [67]. Both shearography and speckle interferometry are strongly affected by environmental disturbances, which limit their application in industrial processes.

**Figure 6:**
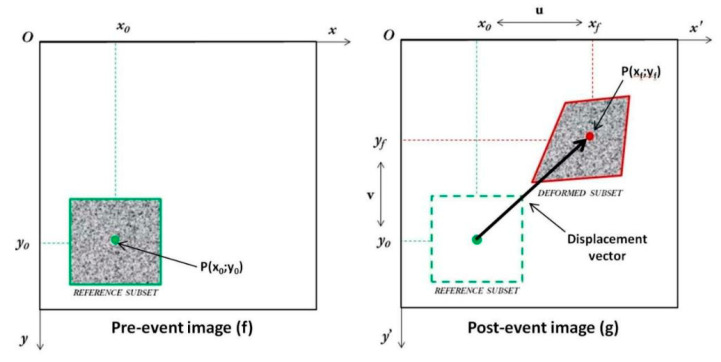
Scheme of DIC basic principle [70].

In the DIC technique, the random speckle pattern is done by manually spraying the surface of the component. DIC method tracks surface displacements in a sequence of digital images under the application of external loads. In this way the DIC allows also to locally capture deformation originated by material inhomogeneities [68]. In particular, in the post-processing phase, a virtual mesh is generated on the surface of the component whose main parameters are the subset size and the step size. The surface of the component is divided into squared subsets whose dimension is defined by the subset size. With the step size parameter, the distance between the subset centers, i.e., the shift between subsets, is assumed. Generally, each subset should contain at least three speckles and step size should be at least 1/3 of the subset size [69]. Both the step size and the subset size are measured in pixels. As shown in Figure 6, taken from [70], subset displacements are sequentially tracked with reference to their center.

Indeed, DIC assumes that the deformation within the subset is constant. The position of each subset is determined in each digital image by minimizing a correlation function. Several correlation functions have been proposed in the literature and the reader can refer to [69] for a comprehensive description.

Montesano [71] adopted the DIC technique to validate an orthotropic damage model based on empirical material parameters for triaxially braided composites subjected to fatigue loads. In particular, a strong correlation between the measured and the predicted strains was observed at various cyclic intervals. This confirmed the possibility to use the measured strain fields to compute scalar damage variables [11]. Even though not explicated, a damage index for optical methods can be written as:

(8) $$D_{ij}=1-\frac{\varepsilon_{ij,0}}{\varepsilon_{ij}}$$

where $\varepsilon_{ij,0}$ and $\varepsilon_{ij}$ are the deformations in the undamaged and damaged state, with i and j the directions of measurement. The DIC is among the most promising techniques for the nondestructive characterization of composites [72,73]. In the literature, its application at the microscale of carbon fiber composites has been also investigated [74]. After determining the optimal DIC parameters through numerical simulations, the Authors investigated images acquired through scanning electron microscope during compression tests on small size specimens. At low magnification, the fibers acted in a speckle pattern and the DIC was able to correctly capture the strain fluctuations due to the presence of matrix-rich and fiber-rich zones. 

## 6. Thermography

The thermography technique is a well-established non-contact technique, which inspects the health state of a structure through temperature gradients. An infrared camera is generally used to measure the temperature gradients, which can be induced both by mechanically loading the structure and by applying thermic impulse. Accordingly, thermography is distinguished in passive and active. The infrared camera records the temperature map of the inspected surface as it evolves in time. The heating transfer to the rest of the component depends on the local thermal properties. A damage, such as a delamination, slows the heat diffusion and, at the infra-red camera, appears as a hot spot. Thermography is a rather expensive technique, but it is able to investigate rather quickly large regions. Further, it provides little information about the volumetric distribution of damage [20].

In the passive thermography, the macroscopical thermomechanical response of a component is analyzed through the application of mechanical loads. From the thermodynamics principles, the heat conduction equation can be written as [75]:

(9) $$\rho c\dot{T}+div\left(q\right)=\Delta +r+\rho T\frac{\delta^{2}\Psi }{\delta T\delta \varepsilon }∶\dot{\varepsilon }+\rho T\frac{\delta^{2}\Psi }{\delta T\delta V}∶\dot{V}$$

where ρ is the material density, c the specific heat capacity, T the temperature, q the heat influx vector, Δ the intrinsic dissipation, r the external heat source, Ψ the Helmotz free energy, ε the strain tensor and V the internal variables vector which complete the description of the thermodynamic state. $\rho T\frac{\delta^{2}\Psi }{\delta T\delta \varepsilon }∶\dot{\varepsilon }$ and $\rho T\frac{\delta^{2}\Psi }{\delta T\delta V}∶\dot{V}$ are the thermo-mechanical coupling terms. Usually, temperature gradients are low, which allows to neglect the coupling between temperature and the internal variables other than the strain tensor and to neglect the variations of ρ and c.

In the elastic regime of an isotropic material, where the dissipation Δ is negligible as well as the heat flux vector compared to the thermoelastic production of heat, Equation (9) can be simplified to [76]:

(10) $$\Delta T=-K_{m}T_{0}\Delta \left(\sigma_{x}+\sigma_{y}+\sigma_{z}\right)$$

where $K_{m}$ is the thermo-elastic coefficient and $\Delta \left(\sigma_{x}+\sigma_{y}+\sigma_{z}\right)$ the variation of the stress state of the material. Equation (10) well interprets the thermomechanical response of a loaded component even if made of anisotropic composites. When subjected to mechanical loading, the temperature of a solid medium decreases (respectively increases) proportionally to the applied tensile (respectively compressive) load [77,78]. The thermo-mechanical coupling is the result of the volume increase when a component is tensile loaded. The deviation from the linear trend of the thermo-elastic coefficient $K_{m}$ indicates the damage initiation [79]. Thanks to this peculiar characteristic, the infrared thermography has been widely applied for the nondestructive monitoring of damage evolution in mechanical tests of composites.

**Figure 7:**
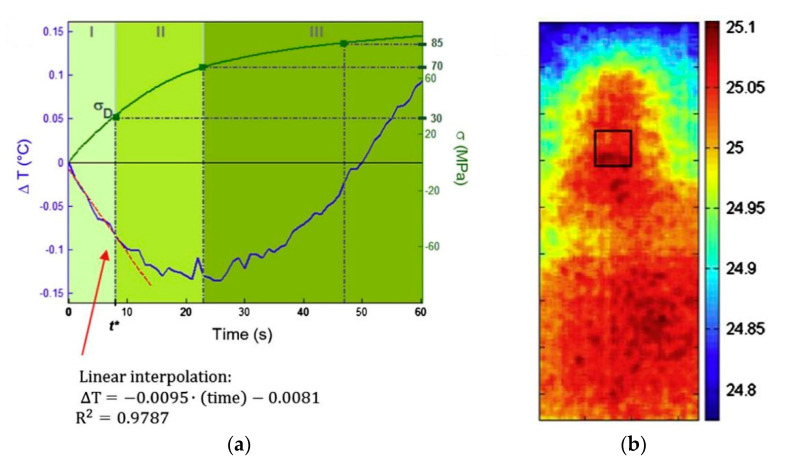
(**a**) Temperature trend and stress trend of the [+45°, −45°]_s_ specimen. Deviation from linearity indicates the damage onset, specifically at time t* and stress $\sigma_{D}$; (**b**) thermal map of the specimen at t*. The black box is the region where the average temperature is calculated [79].

Damage evolution in static tests has been investigated with passive thermography. In [79], the Authors performed quasi-static tests on glass-fiber reinforced specimens. Three configurations were retained: unidirectional laminate loaded in the fiber direction, unidirectional laminate transversely loaded, and symmetric laminates with stacking sequence [+45°, −45°]_s_. The temperature profiles showed three distinct phases. An example, taken from [79], is reported in Figure 7.

In the first phase a linear decrement of the surface temperature with the applied stress is observed, in accordance with the Kelvin’s law, Equation (10). The second phase is characterized by a nonlinear decrement of the temperature, as the damage has onset. At the end of the second phase a minimum in the temperature decrease is reached. The minimum might also represent the high cycle fatigue limit of the material [80], while the true endurance limit is represented by the damage initiation stress. However, the definition of a fatigue limit for composites is still discussed [81]. Finally, the third phase is characterized by an almost linear increment of the temperature. Indeed, as the damage proceeds, the mechanical energy stored in the material is released leading to an increment of the temperature. The slope of the third-phase temperature profile seems correlated with the damage development: the more the damage expands before the final fracture, the higher the slope of the temperature increase [79].

Based on similar considerations, Harizi et al. [77] proposed a damage parameter for quantitatively assess the damage severity through thermography investigations. In particular, the nonlinearity of the stress–temperature relationship is exploited and the damage index is defined as:

(11) $$D=1-\frac{K_{m,0}}{K_{m}}$$

where $K_{m,0}$ and $K_{m}$ are the initial and current thermo-elastic coefficients. In [77,82], the thermo-elastic coefficient was calculated on the average temperature trend of the whole specimen. Indeed, in the investigated cross-ply laminates, the matrix cracking of off-axis plies was rather extended in the specimen length. The thermo-elastic coefficient can be also adopted as damage severity parameter in case of localized damage, by properly restricting the area inspected with the infrared camera. However, it is worth noticing that, as such, the parameter is not able to account for the material anisotropy. Further, such damage evaluation appears limited to the particular case of the stepwise static tensile loading [11].

As plastic reinforced composites are not highly dissipative, monitoring of damage evolution through temperature variations has been preferably performed in fatigue tests. Montesano et al. [78,83] have shown the ability of the infrared thermography technique in evaluating the damage states in textile composite laminates under fatigue. Temperature profiles captured during cycling testing directly well correlated with corresponding stiffness degradation profiles, thus proving the accuracy of thermography as a fatigue damage metric. In a following work [80], infrared thermography was adopted in stepwise fatigue tests to non-destructively investigate the existence of a high cycle fatigue limit in triaxially braided carbon fiber laminates. One single stepwise test was sufficient to determine the fatigue limit, which resulted in very good agreement with standard fatigue characterization, thus extending the application of the method proposed by Risitano [84] for metals. Similar result was obtained by Jegou et al. [85] on short fiber reinforced plastics, who also calculated the dissipation (Δ in Equation (9)) produced within one cycle and then estimated the Wöhler curve from the stepwise test.

In the active thermography, an external source of heat is provided to the component, in order to induce a rise of the surface temperature. Usually, halogen lamps are adopted, in pulsed or continuous mode. Laser allows to locally heat the component. Heat can also be internally induced through ultrasounds or eddy currents [86]. Due to the altered thermal properties in correspondence of the damage, active thermography has been proved successful in damage detection, particularly in the case of surface flaws [87]. Garnier et al. [20] showed that the active thermography is extremely efficient compared to other techniques, even in case of components with nonplanar geometries. Harizi et al. [88] have also proposed a damage index based on active thermography measurements, performed after tensile tests at different maximum stresses. The damage index concerns the cooling rate of the specimen temperature, after a heating process of 30 s with a sinusoidal halogen excitation. The Authors observed an increase in the cooling rate with the increasing applied load. Therefore, the damage variable was defined as:

(12) $$D=1-\frac{{\dot{T}}_{0}}{\dot{T}}$$

where, ${\dot{T}}_{0}$ and $\dot{T}$ are the initial and current cooling rate. However, the damage variable was not particularly sensitive to the material damage, as in correspondence of the final fracture, it barely reached the 30%. Further, it should be noticed that the temperature is not an intrinsic material property, as well as its rate of change in time. In particular, the cooling process also depends on the geometry and on the thermal boundary conditions. As a consequence, the use of such damage parameters is very limited and not able to properly account for the damaged material response.

Indeed, it should be always kept in mind that the temperature is not an intrinsic material property, as it depends on the specimen geometry and on the thermal boundary conditions. Thermography rather provides a photography of the resulting energetic equilibrium of the sample. Therefore, its application should be focused on the quantification of the intrinsic dissipation Δ, which is associated to the damage evolution [89]. However, as reported in some works [78,85], the hypothesis that the dissipated heat measured with the infrared thermography is due to the intrinsic material dissipation seems supported by experimental evidence. As such, thermography is more like an SHM method rather than a proper NDT.

## 7. Resistivity-Based Measurements

Measurements of the electrical resistance are restricted to conductive composites, which are typically carbon fiber-based laminates. As current flows through the fibers, their rupture causes a decrease in the section current flow, thus resulting in an increment of the electrical resistance. Probes usually made of copper are used to provide the electrical current and the voltage. Two configurations are typical in industrial applications. The two-probe method, where two electrodes are used to provide the current and to measure the voltage drop, and the four-probe method, where the current flows between the external probes and the internal probes are used for voltage measurements. In electrical impedance tomography, measurements of the voltage are repeated on the component, in order to extract information on the position and size of the damage [90].

The sensitivity of the technique is maximum for fiber rupture, as the increase of the electrical resistance in case of such damage is particularly enhanced. Further, the initial resistance, i.e., the resistance in the undamaged and undeformed configuration, in fiber direction is small and a model of parallel resistances can be adopted [91]. Abry et al. [92] have shown that DC current is preferable to AC current for in-plane, in fiber direction measurements. However, the technique is also sensitive to the subcritical matrix cracking damage and to delamination, even though in the considered directions the initial resistance is constantly higher than that in the fiber direction. The in-plane and the through-the-thickness transverse directions can be modelled as a series of resistances. According to Abry et al. [92], through-the-thickness AC current is more suitable for matrix cracking and delamination damage. For delamination detection, the current should be charged in the through-the-thickness direction [93].

The resistivity-based technique is adopted both for the real-time SHM and as offline NDT. Indeed, the deformation of the structure also affects the electrical conductivity of the material [94]. The application of loads results in a linear increase of the resistance until the damage occurs [92]. Thereafter, a nonlinear increment is observed. It is worth noticing that, even though the linear–nonlinear threshold in the resistance increase can be used to define a critical level of warning, a corresponding degradation of the mechanical properties is not necessarily observed. Further, as shown by some Authors [95], in presence of matrix cracks, fibers contact can be favored by the application of loads, which results in decrement of the electrical resistance. In this regard, a further contribution can be provided by the slight reorientation of the fibers in the loading direction [95].

Todoroki et al. [96] have shown that the increase of the electrical resistance is evident also without the application of external loads, if matrix cracking has occurred. In particular, the Authors analyzed cross-ply laminates, highlighting the increase of the resistance due to the formation of matrix cracks in the off-axis laminae. In addition, the change of resistance well correlated with the crack density.

A damage index can be therefore defined as [97]:

(13) $$D=1-\frac{R_{0}}{R}$$

where $R_{0}$ and R are the initial and current electrical resistance, respectively. Seo et al. [97] showed that the measured stiffness and electrical resistance presented a very similar trend of change during fatigue tests of cross-ply and unidirectional carbon fiber laminates. In particular, they observed that the resistance increase replicated very well the progressive increase of strain under the applied cyclic load in the cross-ply laminate. Instead, in the unidirectional laminate, the resistance increased more scattered and with limited accordance with the strain. This different behavior occurs particularly since the increase of the electrical resistance is not inversely proportional to the residual electrical section. The current can also flow in the in-plane transverse direction due to the contact between fibers, thus leading to a lower increase of the resistance with respect to the theoretical prediction [98]. In the cross-ply laminates under fatigue loading, matrix cracking of off-axis plies firstly occurs, preventing the current from flowing in the transverse direction. The different damage mechanism of the unidirectional laminate instead allows the transversal current flows, thus leading to more scattered measurements. The correlation between the damage state of the material and the resistance measurement can thus result critical.

The resistivity-based technique can be also applied to glass fiber composites, provided that the matrix is filled with conductive particles, usually made of carbon. As an alternative, the measurement of the dielectric response in composite laminates has been recently proposed by Vadlamudi et al. [99]. The technique intends to observe and model the variation in the dielectric response as a function of the damage evolution in order to characterize the current material state. This interesting idea is based on the observation that the presence of cracks, voids etc., increases the presence of internal surfaces thus altering the capacitive properties of the material [100,101]. In glass fiber [+45°, −45°] laminates, trends similar to those obtained with infrared thermography (Cf. Section 6) were reported. An initial linear increase of the real part of the electric permittivity with the applied strain was observed as result of the interfacial polarization during the formation of micro-cracks. A nonlinear second phase then followed where a saturation of the real electrical permittivity is reached. In the third phase, a decrease of the permittivity is observed. The technique thus intends to characterize the current material state and has been demonstrated to be able to well identify the different phases of damage progression, even though a quantitative damage index is not explicitly proposed.

## 8. Conclusions

In the present paper, the NDTs able to provide a quantitative assessment of the damage in laminated composites have been reviewed. In composites, the multitude of damage mechanisms, whose evolution and interaction are still relevant research topics, suggests the need of assessing the damage severity on the basis of the material response in the presence of damage or flaws, rather than on the only extension of the damaged area, as typical in metals. According to the retained NDT, different kinds of material responses are investigated. For the wave methods (ultrasonic technique and Lamb waves-based method), as well as for the vibrational method and for the Detecting Damage Index, the residual elastic properties are evaluated. Other techniques, such as the thermography or the resistivity-based technique, involve other aspects of the material response in presence of damage or defects. It is worth noticing that reliable models which correlate the residual strengths of the composite to the damage state are still required [7].

Table 1 summarizes the reviewed NDTs, focusing on the significance of the damage index retained with each technique. Advantages and limitations are thus drawn with respect to the technique and to the correlated damage index.

The following conclusions can be highlighted:

1. Only a limited number of techniques are able to mechanically characterize the residual elastic properties. These are the wave-based methods, the local vibrational analysis, the Detecting Damage Index, and the optical methods, with particular reference to the DIC. In thermography, the thermo-elastic coupling also allows mechanical considerations, even though the material direction dependency is not retained.
2. In ultrasonics and acousto-ultrasonics, time-of-flight measurements are controversial when the phase velocity and the group velocity are severely distinct. This particularly limits the application of such techniques to angle-ply laminates. Further, it is worth highlighting that the assessment concerns all the material comprised between the transducers. However, the use of acoustic emission analysis methodologies, such as the evaluation of SWFs in the frequency domain, seems extremely promising.
3. Local vibrational analysis and the Detecting Damage Index are both able to account for the material direction dependency and the assessment concerns only the local material properties. The main drawback of both techniques is that they can be applied only to planar or partially curved surfaces. Due to its novel characteristics, the local vibrational analysis also requires further investigations and validations. Instead, the Detecting Damage Index is characterized by an extended preliminary experimental campaign.
4. The optical methods are able to capture local deformations. More rapid displacement gradients are observed in correspondence of material inhomogeneities, thus allowing a local material characterization. External load is necessary, which might limit their application on real-world component. Optical methods are also characterized by elevated costs and the necessity of a speckle pattern.
5. Thermography and resistivity-based techniques can inspect very large areas in a short time. The non-mechanical physics of these techniques however limits their application for quantitative assessment of the damage. In particular, the dependency of the material properties on the direction cannot be retained. Further, for the thermography, it is worth to highlight that temperature is not a material property and thermo-mechanical measurements should refer to the intrinsic dissipation which is associated to the damage process.

It is the authors’ believe that NDTs are to be properly combined with the material behavior models in order to define damage tolerant design strategies for composites. Such combination would thus lead to safer and more cost-effective composite structures.

## Figures and Tables

**Table 1:** Summary of the investigated NDTs and the related damage indexes.

NDT	Damage Parameter	Advantages	Limitations	Ref.
Ultrasonics	$D_{ij}=1-\frac{C_{ijkl}}{C_{ijkl,0}}$	Damage location and size identification	Quantitative assessment limited to cross-ply laminates Assessment related to the material comprised between the transducers	[21,22,23,24,25,26]
Acousto-ultrasonics	$D=1-\frac{E_{11}}{E_{11,0}}$	Quick investigation of large areas Correlation with crack density in the matrix	Quantitative assessment limited to cross-ply laminates Assessment related to the material comprised between the transducers	[17,18,19,20,21,22,23,24,25,26,28,29,30,31,32]
Local Vibrational Analysis	$D_{ij}=1-\frac{E_{ij}}{E_{ij,0}}$	Local material assessment Material direction dependency Easiness of testing	Limited to planar region of the component	[33,34,35,36,37,38,39,40,41,42,43]
Detecting Damage Index	$D_{ij}=1-\frac{E_{ij}}{E_{ij,0}}$	Local material assessment Material direction dependency Easiness of testing	Limited to planar region of the component Extensive preliminary campaign	[44,45,46,47,48,49,50,51]
DIC	$D_{ij}=1-\frac{\varepsilon_{ij,0}}{\varepsilon_{ij}}$	Local material assessment Material direction dependency	Required speckle pattern Elevated costs	[52,53,54,55,56,57,58,59,60,61]
Thermography	$D=1-\frac{K_{m,0}}{K_{m}}$ (passive) $D=1-\frac{{\dot{T}}_{0}}{\dot{T}}$ (active)	Quick investigation of large areas All component geometries	Unable to account for the material direction dependency	[62,63,64,65,66,67,68,70,71,72,73,74,75,76,77]
Resistivity	$D=1-\frac{R_{0}}{R}$	Both real-time and offline monitoring All component geometries	Limited to conductive materials Mechanical properties degradation not necessarily correlated	[78,89]

## Data Availability

No new data were created or analyzed in this study.
